# Ten years of monitoring malaria trend and factors associated with malaria test positivity rates in Lower Moshi

**DOI:** 10.1186/s12936-021-03730-1

**Published:** 2021-04-20

**Authors:** Nancy A. Kassam, Robert D. Kaaya, Damian J. Damian, Christentze Schmiegelow, Reginald A. Kavishe, Michael Alifrangis, Christian W. Wang

**Affiliations:** 1 Kilimanjaro Christian Medical University College (KCMUCo), P.O. Box 2240, Moshi, Tanzania; 2 School of Public Health and Family Medicine, University of Cape Town, Cape Town, South Africa; 3 Centre for Medical Parasitology, Department of Immunology and Microbiology, University of Copenhagen, Copenhagen, Denmark; 4 Department of Infectious Diseases, Copenhagen University Hospital (Rigshospitalet), Copenhagen, Denmark

**Keywords:** Malaria, test positivity rates, High altitude malaria, Tanzania

## Abstract

**Background:**

High altitude settings in Eastern Africa have been reported to experience increased malaria burden due to vector habitat expansion. This study explored possible associations between malaria test positivity rates and its predictors including malaria control measures and meteorological factors at a high-altitude, low malaria transmission setting, south of Mount Kilimanjaro.

**Methods:**

Malaria cases reported at the Tanganyika Plantation Company (TPC) hospital’s malaria registers, meteorological data recorded at TPC sugar factory and data on bed nets distributed in Lower Moshi from 2009 to 2018 were studied. Correlation between bed nets distributed and malaria test positivity rates were explored by using Pearson correlation analysis and the associations between malaria test positivity rates and demographic and meteorological variables were determined by logistic regression and negative binomial regression analyses, respectively.

**Results:**

Malaria cases reported at TPC hospital ranged between 0.48 and 2.26% per year and increased slightly at the introduction of malaria rapid diagnostic tests. The risk of testing positive for malaria were significantly highest among individuals aged between 6 and 15 years (OR = 1.65; 1.65 CI = 1.28–2.13; *p* = 0.001) and 16–30 years (OR = 1.49; CI = 1.17–1.89; *p* = 0.001) and when adjusted for age, the risk were significantly higher among male individuals when compared to female individuals (OR = 1.54; 1.00–1.31; *p* = 0.044). Malaria test positivity rates were positively associated with average monthly minimum temperatures and negatively associated with average monthly maximum temperatures (incidence rate ratio (IRR) = 1.37, 95% confidence interval (CI) = 1.05–1.78, *p* = 0.019 and IRR = 0.72, 95% CI = 0.58–0.91, *p* = 0.005, respectively). When analysed with one month lag for predictor variables, malaria test positivity rates were still significantly associated with average monthly minimum and maximum temperatures (IRR = 1.67, 95% CI = 1.28–2.19, *p* = 0.001 and IRR = 0.68, 95% CI = 0.54–0.85, *p* = 0.001, respectively). Average monthly rainfall and relative humidity with or without a one month lag was not associated with malaria test positivity rates in the adjusted models. Explopring possible associations between distribution of long-lasting insecticidal nets, (LLINs) and malaria test positivity rates showed no apparent correlation between numbers of LLINs distributed in a particular year and malaria test positivity rates.

**Conclusion:**

In Lower Moshi, the risk of being tested positive for malaria was highest for older children and male individuals. Higher minimum and lower maximum temperatures were the strongest climatic predictors for malaria test positivity rates. In areas with extensive irrigation activity as in Lower Moshi, vector abundance and thus malaria transmission may be less dependent on rainfall patterns and humidity. Mass distribution of LLINs did not have an effect in this area with already very low malaria transmission.

**Supplementary Information:**

The online version contains supplementary material available at 10.1186/s12936-021-03730-1.

## Background

More than 229 million malaria cases occurred globally in 2019 of which 94% occurred in sub-Saharan Africa (SSA) [1]. Tanzania contributes 3% of malaria burden in the malaria endemic African region and was among the countries which contributed the highest to global malaria deaths (5%) [1]. Meteorological factors including temperature, rainfall and humidity largely affect distribution of malaria through its impact on vector development, distribution and abundance [2–7]. Malaria vectors, the *Anopheles* mosquitoes, develop and survive better at an optimal temperature of about 27 ℃ in a range of 17–35 ℃ [8, 9]. At optimal temperatures the parasites’ life cycle within *Anopheles* is shortened [8], number of blood meals taken and the number of mosquito eggs laid are increased and, therefore, the density of the mosquito population is accelerated [9]. On the other hand, temperatures higher than the optimal temperature negatively affect mosquito survival, and thus, malaria transmission.

Malaria transmission intensity is also altitude dependent because temperature decrease with increasing altitude [10]. Beyond 2400 m above sea level the temperature is too low to support mosquito survival and malaria transmission [11, 12]. Humidity as well affects malaria transmission through its effect on adult mosquito activity and length of survival as they become more active and survive longer in a high humidity environment as compared to low humidity environment [13].

Rainfall also affects malaria transmission as *Anopheles* mosquitoes breed in stagnant water that pools following rainfalls and breed more often in fresh rain water than in polluted water [14]. However, too much rainfall flushes off breeding sites making malaria transmission lower than expected [15]. Malaria transmission is usually preceded by a vector breeding and development period which also depends on climatic variables, mainly temperature and rainfall. Thus, there is a considerable lapse between the period of vector development and malaria transmission with a maximum significant correlation of one month lag period [16].

Increased agricultural activities such as development of irrigation channels creates mosquito breeding sites and hence increased malaria transmission [2, 17, 18]. However, irrigation schemes may also cause less malaria transmission in areas with previously known stable transmission, proposedly due to better living conditions in communities with growing wealth and attraction of mosquitoes that are less able to transmit malaria [19].

Malaria control measures are claimed to have significantly lowered the global malaria prevalence from the year 2000 to 2015 [20–22], but this decline slowed in rate after 2015 [1]. Additionally, global warming has impacted malaria transmission through expansion of vector habitat as temperature has increased in high altitudes which were previously malaria free settings thus, highland areas are at an increased risk for malaria transmission [7, 23–25]. Exploring trends of malaria infections and factors that may affect malaria transmission in areas prone to malaria elimination, such as highland areas of Tanzania are needed to understand the progress towards elimination and the potential for rebound. The aim of this study was to retrospectively describe 10 years of malaria cases reported at Tanganyika Plantation Company (TPC) hospital, a high-altitude malaria pre-elimination zone of Lower Moshi in Kilimanjaro, from 2009 to 2018 and explore possible associations between malaria test positivity rates and meteorological variables.

## Methods

### Design and settings

The study was conducted at TPC Hospital in Lower Moshi (3021′S, 37,020′E), located 10 km from Moshi municipality, and about 800 m above sea level, south of Mount Kilimanjaro in the north-eastern Tanzania. Most of the population in the area is engaged in irrigation of rice and sugarcane activities which provide important breeding sites [17, 26] for *Anopheles arabiensis,* the predominant malaria vector in the area [27]. Lower Moshi is an area of low malaria transmission intensity with malaria prevalence less than 0.1% [17, 26] and entomological inoculation rate (EIR) of 0.54 infective bites/person/year [28]. At TPC hospital the standard diagnostic tool for malaria was microscopy until 2012, thereafter rapid diagnostic tests (RDTs) were used for diagnosis and microscopy was used to quantify parasitaemia and control parasite clearance following treatment.

### Data sources

Data used in this study were obtained from the TPC hospital’s haematology laboratory registers on malaria cases from January 2009 to January 2017; the Health Management Information System (HMIS) for the period between February 2017 to November 2017; and the Micro-Health Initiative (MHI) register on routine malaria testing between December 2017 and December 2018. Data for the total daily number of individuals tested for malaria, number of malaria-confirmed cases and the type of malaria diagnostic tool used were extracted.

Meteorological data from 2009 to 2018 recorded at the TPC estate meteorological station included daily rainfall in millimetres, minimum and maximum relative humidity, and minimum and maximum temperature. Data on numbers of long-lasting insecticidal nets (LLINs) distributed in the area for the study period were obtained from the national malaria control programme (NMCP) focal team for Moshi District Council. All malaria test data available for a 10-year period (January 2009-December 2018) was included in the analysis. All records that did not have complete information, e.g., if the testing tool was not indicated were excluded.

### Variables and definitions

The predictor variables were age, gender, rainfall, temperature, humidity and bed nets distributed and the outcome variable was malaria test positivity rates. Rainfall data was analysed as total monthly rainfall; temperature was analysed as average monthly minimum temperature and average monthly maximum temperature; humidity was analysed as average monthly minimum relative humidity and average monthly maximum relative humidity. Parasitaemia was categorised as low (< 1000 parasites/µl), moderate (1000–4999 parasites/µl), high (5000–99,999 parasites/µl) and hyperparasitaemia (≥ 100,000 parasites/µl).

### Statistical analysis

Data were analysed using Stata version 14·0 (StataCorp. 2015. Stata Statistical Software: Release 14. College Station, TX: StataCorp LP). Pearson’s correlation statistic was used to measure the strength of linear relationship between malaria test positivity rates and bed nets distributed. Logistic regression analysis was used to determine the associations between malaria test positivity rates and characteristics of individuals tested for malaria. Univariate and multivariate negative binomial regression analyses were used to determine associations between malaria test positivity rates and meteorological variables. Time series negative binomial regression analysis was also used to adjust for the lagged association between malaria test positivity rates and meteorological variables. All meteorological variables analysed in the univariate analysis were included in the multivariate analysis as they are all important predictors of malaria transmission and for all variables, the degrees of collinearity were found to be less than 45%. A *p*-value < 0.05 was considered statistically significant in all tests.

## Results

### Characteristics of individuals tested for malaria at TPC hospital from 2009 to 2018

A total of 73,694 individuals were tested for malaria at TPC hospital from 2009 to 2018 of these 1.21% (n = 895) tested positive for malaria. Individuals aged between 16 and 30 years represented the population most tested for malaria (n = 19,158; 26.00%) and more female individuals were tested (n = 38,351; 52.04%). Parasitaemia was recorded for 872 (97%) individuals out of 895 malaria positive cases and nearly 40% (n = 337) had moderate parasitaemia (Table 1).

**Table 1:** Characteristics of individuals tested for malaria at TPC hospital from 2009 to 2018 N = 73,694

Variable	n	%
Age category		
0–5	13,633	18.50
6–15	10,244	13.90
16–30	1158	26.00
31–45	17,417	23.63
46–65	9003	12.22
66 +	4239	5.75
Median age in years (range)	26 (0–110)	
Gender		
Female	38,351	52.04
Male	35,343	47.96
Malaria test		
Positive	895	1.21
Negative	72,799	98.79
Parasitaemia (n = 872)		
Low parasitaemia	309	35.44
Moderate parasitaemia	337	38.65
High parasitaemia	216	24.77
Hyperparasitaemia	10	1.15
Geometric mean (SD)	2035 (4.84)	

*SD* Standard deviation

### Trends of annual proportion of malaria cases reported from 2009 to 2018

**Fig. 1:**
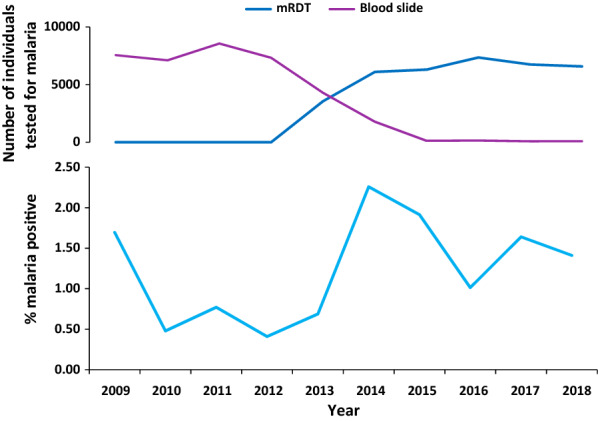
Proportion of malaria positive cases tested by either microscopy or RDTs between 2009 and 2018 at TPC Hospital

Trend of malaria and numbers of individuals tested for malaria using either microscopy or RDTs are depicted in Fig. 1, where the upper graph shows the number of individuals tested by either microscopy or RDTs and the lower graph shows the annual proportion of malaria cases among individuals tested. A total of 73,694 individuals were tested for malaria using either microscopy (n = 37,072) or RDTs (n = 36,622) during the 10-year study period (2009–2018). The total number of tested individuals (6427–8563/year) did not differ markedly between the years 2009 and 2018. Of those tested, 895 (1.21%) were positive for malaria (Fig. 1 and Additional file 1).

Overall, the yearly proportions of malaria cases were low, fluctuating between 0.48 and 2.26% with a relatively lowest period from 2010 to 2013 and a small increase in 2014 with the highest number of recorded cases. From 2015 to 2018 the proportions of recorded malaria cases were between 1.01 and 1.91% (Fig. 1).

### Malaria test positivity rate and rainfall pattern

**Fig. 2:**
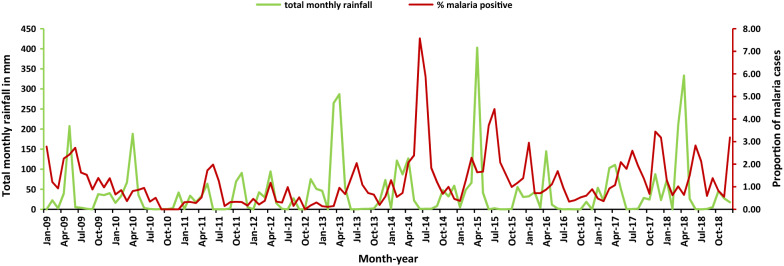
Monthly proportion of malaria cases among individuals attending the TPC Hospital from 2009 to 2018 and total monthly rainfall for Lower Moshi

The rainfall pattern for Lower Moshi generally peaked twice every year, with a heavy rainy season within the months of March to May, and another rainy season with less rain within the months of October to December (Fig. 2 and Additional file 1). After 2012, during the heavy rainy seasons malaria test positivity rates increased and peaked either towards the end of the rainy season or immediately after the rains. During the second rainy seasons, malaria test positivity rates did not seem to follow the rain pattern for most of the years.

### Malaria test positivity rates and bed net distribution

Large numbers of LLINs were distributed to households in the villages in Lower Moshi in 2014 (n = 21,068) and 2016 (n = 56,913) (Additional file 1). In the other years, up to 3,680 LLINs were distributed through all health facilities within Lower Moshi. However, bed net increase did not seem to associate with malaria test positivity rates (Pearson’s correlation coefficient (r) = 0.109; *p* = 0.765). Data on bed net presence and use in the respective households were not available.

### Associations between malaria test positivity and characteristics of individuals tested for malaria at TPC hospital

In the adjusted model, the risk of testing positive for malaria was significantly higher among individuals aged between 6–15 years (OR = 1.65; CI = 1.28–2.13; *p* = 0.001) and 16–30 years (OR = 1.49; 1.17–1.89; *p* = 0.001) when compared to individuals aged between 46–65 years. When adjusted for age, the risk of testing positive for malaria was significantly higher among male individuals when compared to female individuals (OR = 1.54; 1.00–1.31; *p* = 0.044) (Table 2). Strikingly, children under 5 years were not at higher risk of testing positive.

**Table 2:** Associations between malaria test positivity and characteristics of individuals tested for malaria at TPC hospital

Variable	COR (95% CI)	*p*-value	AOR (95% CI)	*p*-value
Age categories				
0–5	0.78 (0.59–1.03)	0.085	0.78 (0.59–1.03)	0.083
6–15	1.64 (1.27–2.12)	0.001	1.65 (1.28–2.13)	0.001
16–30	1.48 (1.17–1.88)	0.001	1.49 (1.17–1.89)	0.001
31–45	1.13 (0.88–1.45)	0.351	1.13 (0.88–1.45)	0.329
46–65	1		1	
66 +	1.03 (0.72–1.45)	0.884	1.32 (0.72–1.48)	0.858
Gender				
Female	1		1	
Male	1.12 (0.98–1.28)	0.083	1.54 (1.00–1.31)	0.044

*COR* crude odds ratios, *AOR* adjusted odds ratios

### Associations between malaria test positivity rates and meteorological variables without time lag

The association between malaria test positivity rate and meteorological variables without the introduction of a time lag phase are shown in Table 3. The range of average monthly minimum and maximum temperatures were 16.2–22.7 and 27.2–35.7 °C, respectively (Additional file 1). In the adjusted analysis, malaria test positivity rates were significantly associated with average monthly minimum temperatures and negatively associated with average monthly maximum temperatures (IRR = 1.37; CI = 1.05–1.78; *p* = 0.019 and IRR = 0.72; CI = 0.577–0.91; *p* = 0.005, respectively). Regarding relative humidity, the crude malaria test positivity rates were significantly associated with average monthly maximum relative humidity (IRR = 1.06; CI = 1.01–1.10; *p* = 0.009) and average monthly minimum relative humidity (IRR = 1.03; CI = 1.01–1.06; *p* = 0.007), but not in the adjusted model. There was no significant association between rainfall and malaria test positivity rates, both in the crude and the adjusted models (Table 3).

**Table 3:** The association between malaria test positivity rates and temperature, humidity, and rainfall in Lower Moshi from 2009 to 2018

Variable	Crude analysis IRR (95% CI)		*p*-value	Adjusted analysis IRR (95% CI)	*p*-value
Total monthly rainfall	1.00 (1.00–1.001)		0.464	1.00 (0.99–1.00)	0.217
Average monthly max temp	0.87 (0.81–0.94)		0.001	0.72 (0.58–0.91)	0.005
Average monthly min temp	0.92 (0.82–1.02)		0.115	1.37 (1.05–1.78)	0.019
Average monthly max RH	1.06 (1.01–1.10)		0.009	1.00 (0.92–1.10)	0.918
Average monthly min RH	1.03 (1.01–1.06)		0.007	0.99 (0.95–1.03)	0.583

*RH* Relative Humidity, *IRR* incidence rate ratio, *CI* confidence interval, *max *maximum, *min *minimum, *temp* temperature

### Associations between malaria test positivity rates and meteorological variables with a one month lag phase

The association between malaria test positivity rates and meteorological variables was estimated with one month lag for the predictor variables (Table 4). In the adjusted analysis, malaria test positivity rates were significantly associated with average monthly minimum temperatures (IRR = 1.67; CI = 1.28–2.19; *p* = 0.001) and negatively associated with average monthly maximum temperatures (IRR = 0.68; CI = 0.54–0.85; *p* = 0.001). Malaria test positivity rates were significantly associated with average monthly maximum relative humidity (IRR = 1.07; CI = 1.03–1.11; *p* = 0.002) and average monthly minimum relative humidity (IRR = 1.03; CI = 1.01–1.06; *p* = 0.007) in the crude analysis. However, in the adjusted model, malaria test positivity rates had no association with rainfall and relative humidity.

**Table 4:** The association between malaria test positivity rates and one month lag temperature, humidity, and rainfall in Lower Moshi from 2009 to 2018

Variable	Crude analysis IRR (95% CI)	*p*-value	Adjusted analysis IRR (95% CI)	*p*-value
Total monthly rainfall	1.00 (1.00–1.003)	0.554	1.00 (0.99–1.00)	0.212
Average monthly max temp	0.92 (0.85–0.99)	0.030	0.68 (0.54–0.85)	0.001
Average monthly min temp	1.04 (0.93–1.17)	0.458	1.67 (1.28–2.19)	0.001
Average monthly max RH	1.07 (1.03–1.11)	0.002	0.99 (0.92–1.09)	0.974
Average monthly min RH	1.03 (1.01–1.06)	0.007	0.99 (0.95–1.03)	0.626

*RH* Relative Humidity, *IRR* incidence rate ratio, *CI* confidence interval, *max *maximum, *min *minimum

## Discussion

Climate changes, increased use of vector control measures, better testing tools, more effective treatment with anti-malarial drugs and improved socio-economic status is changing the epidemiology of malaria [22, 29].

In Lower Moshi, malaria transmission is low (annual malaria prevalence < 0.1%), but malaria cases are seen throughout the year with an increase in number of cases around the end of the rainy seasons or shortly after. Most likely, irrigation activities are one of the main drivers of persistent malaria transmission during the dry seasons in Lower Moshi as also supported by other studies [17, 26]. The findings of this study showed that malaria cases reported at TPC Hospital in Lower Moshi between 2009 and 2018 were few. Cases were lowest from the year 2010 to 2013 until a small increase in 2014 followed by a slight decline until 2018. The small increase in the number of malaria positive cases in 2014 may be accounted for by the introduction of RDTs, which compared to microscopy is a more sensitive and rapid diagnostic tool [30], but may also introduce some false positive results [31]. On the contrary, other studies have reported large increases in number of malaria positive cases following the introduction of RDTs [32, 33]. Lack of this observation in the current study could be due to the accuracy of blood slide test performance due to the presence of qualified staff and performance of quality controls. Unfortunately, there was too little overlap in the usage of RDTs and microscopy to do a sensitivity analysis in the current study.

Similar findings, though on a greater scale, have been reported in other settings in Tanzania with different malaria transmission intensities where the total number of cases increased from the year 2012 to 2015 followed by a slight reduction in number cases reported in 2016 and 2017 [34, 35]. These findings are partly contrary to what has been reported on the overall trends of malaria in sub-Saharan Africa, where the number of malaria cases showed a significant decline from the year 2000 to 2015 [1, 20, 21] and continued to decline, though, at a much-slower rate after 2015[1]. The inconsistency may be caused by difference in time points when control measures were introduced in the different parts, variations in weather conditions and/or other factors which may contribute to local variations.

Studies have showed that malaria control measures, particularly LLINs, have significantly averted malaria cases in sub-Saharan Africa since the year 2000 to 2015 [21, 36, 37]. In this study, despite immense distribution of LLINs during 2014 and 2017 there was no effect on the numbers of malaria positive cases reported at TPC Hospital. This could probably be due to weather conditions favourable for mosquito population growth at the time, especially moderate prolonged rainfall (Fig. 2), or due to irrational use of bed nets [38, 39]. However, the possible impact of LLINs on malaria incidence is in this particular setting of Lower Moshi also difficult to assess, as the number of malaria cases is already very low. Further studies on LLINs coverage, ownership and usage are warranted to better understand how this affect the malaria trend in the study area.

In this study, the risk of testing positive for malaria was significantly higher among individuals in the age groups 6–30 years when compared to individuals aged between 46 and 65 years. Two different studies conducted in Kisumu Kenya and Gabon also reported similar findings, and showed higher malaria prevalence among children older than 5 years of age [40, 41]. As previously suggested, this is probably due to lower utilization of insecticide treated bed nets (ITNs) by older children [42]. Another study also found significantly higher malaria test positivity among individuals aged between 11 and 20 years [43]. In the present study the risk of testing positive for malaria was not significantly high among individuals aged above 30 years. This is probably due to development of clinical immunity caused by many years of previous exposures in this low transmission area [44]. Furthermore, the risk of testing positive for malaria was significantly higher among male than female individuals which could be due to differences in behaviour related to exposure where women are more likely to use bed nets than men [45].

Meteorological variables including rainfall, temperature and relative humidity did not show any change in Lower Moshi over the course of the ten years investigated, but regular annual patterns which affect malaria transmission temporarily. Temperatures for Lower Moshi showed an annual pattern with average monthly temperatures ranging from 16.2 to 31.4 °C during the cool months of July and August and reached temperatures of 19.7–35.7 °C during the hot months around January to March. Malaria test positivity rates increased with increasing average monthly minimum temperature. These findings are supported by previous studies which have documented that very low temperatures do not support mosquito survival, lowering the malaria transmission [11, 12]. Malaria cases decreased with increasing average monthly maximum temperature when analysed with and without one month time lag. Other studies have shown a similar association that temperature was a strong predictor for malaria incidence when compared to other meteorological variables [4, 16] and it has previously been documented that extremely low temperatures below 17 ℃ and extremely high temperatures above 32 ℃ negatively affects mosquitoes survival [7, 46] and that malaria transmission peaks at 27 ℃ [9, 46].

Rainfall and relative humidity are important factors for malaria transmission as rainfall creates breeding sites that increase vector populations [14] and a humid environment prolongs the lifespan of vectors and increases vector activity [13] where mosquito abundance and malaria transmission are higher during the most humid months of a year [47, 48]. In this study, there was no significant association between malaria incidence and either rainfall or relative humidity. In Lower Moshi, irrigation activities make important breeding sites for mosquitoes during the dry season and probably lowers the effect of rainfall and humidity on malaria transmission as seen in non-irrigation settings. A previous study also documented that, the presence of irrigation activities has dampening effect on the rainfall seasonality of malaria transmission in Tropical Africa [49].

The limitations of this study included lack of access to data on malaria cases imported from other areas and data on other possible malaria transmission confounding factors such as socio-economic status.

## Conclusion

In this study setting with low malaria transmission and extensive irrigation activities, temperatures around the optimal for vector development was the strongest predictor for malaria test positivity rate whereas rainfall, humidity, and mass distribution of LLINs did not seem to influence the number of malaria cases reported. However, further studies on the utilization of bed nets with focus on older children and males is warranted. Highland areas are at an increased risk for malaria transmission due to global warming expanding the potential habitats for malaria vectors and areas with year-round water access for the mosquitoes may be in particular risk.

## Supplementary Information

**Additional file 1**. Malaria, meteorological data and bed nets distributed in Lower Moshi. 1: Monthly malaria cases and total number of individuals tested at TPC hospital, Lower Moshi; 2: Monthly meteorological data recoded at TPC weather station; 3: Bed nets distributed in Lower Moshi from 2009 to 2018.

## Data Availability

Due to ongoing analyses, the supporting data are available from the corresponding author on a reasonable request.

## Abbreviations

AOR: Adjusted Odds Ratio
CI: Confidence Interval
COR: Crude Odds Ratio
EIR: Entomological Inoculation Rate
HMIS: Health Management Information System
IRR: Incidence Rate Ratio
ITNs: Insecticide-Treated bed Nets
KCMUCo: Kilimanjaro Christian Medical University College
LLINs: Long Lasting Insecticidal Nets
MHI: Micro-Health Initiative
RDTs: Rapid Diagnostic Tests
NMCP: National Malaria Control Programme
OR: Odds Ratio
PMI: President’s Malaria Initiative
RH: Relative Humidity
SD: Standard Deviation
SSA: Sub-Saharan Africa
TPC: Tanganyika Plantation Company
WHO: World Health Organization

## References

[1] WHO. World Malaria Report 2020. Geneva, World Health Organization, 2020. Available from: https://www.who.int/teams/global-malaria-programme/reports/world-malaria-report-2020

[2] Kibret S, Wilson GG, Tekie H, Petros B (2014). Increased malaria transmission around irrigation schemes in Ethiopia and the potential of canal water management for malaria vector control. Malar J.

[3] Guo C, Yang L, Ou CQ, Li L, Zhuang Y, Yang J (2015). Malaria incidence from 2005–2013 and its associations with meteorological factors in Guangdong. China Malar J.

[4] Mohammadkhani M, Khanjani N, Bakhtiari B, Sheikhzadeh K (2016). The relation between climatic factors and malaria incidence in Kerman, South East of Iran. Parasite Epidemiol Control.

[5] Darkoh EL, Larbi JA, Lawer EA (2017). A weather-based prediction model of Malaria prevalence in Amenfi West District. Ghana Malar Res Treat.

[6] Kajeguka D, Tarmo S (2017). Meteorological influence in pattern of malaria cases in North-Eastern Tanzania: five years analysis of malaria incidence and climate condition. J Pathol Microbiol.

[7] Kulkarni MA, Desrochers RE, Kajeguka DC, Kaaya R, Tomayer A, Kweka E (2016). 10 Years of environmental change on the slopes of Mount Kilimanjaro and its associated shift in malaria vector distributions. Front Public Health.

[8] Shapiro LLM, Whitehead SA, Thomas MB (2017). Quantifying the effects of temperature on mosquito and parasite traits that determine the transmission potential of human malaria. PLoS Biol.

[9] Christiansen-jucht CD, Parham PE, Saddler A, Koella JC, Basáñez M. Larval and adult environmental temperatures influence the adult reproductive traits of *Anopheles gambiae s.s.* Parasit Vectors. 2015;8:456. 10.1186/s13071-015-1053-5 PMC4573685 26382035

[10] Drakeley CJ, Carneiro I, Reyburn H, Malima R, Lusingu JPA, Cox J (2005). Altitude-dependent and independent variations in *Plasmodium falciparum* prevalence in Northeastern Tanzania. J Infect Dis.

[11] Omumbo JA, Hay S, Goetz S, Snow R, Rogers D (2002). Updating historical maps of malaria transmission intensity in East Africa using remote sensing. Photogramm Eng Remote Sensing.

[12] Bishop RA, Litch JA (2000). Malaria at high altitude. J Travel Med.

[13] Yamana TK, Eltahir EAB (2013). Incorporating the effects of humidity in a mechanistic model of *Anopheles gambiae* mosquito population dynamics in the Sahel region of Africa. Parasit Vectors.

[14] Omolade OO, Adetutu SA (2018). Oviposition and breeding water sites preferences of mosquitoes within Ojo area, Lagos State. Nigeria J Sci Tech Res.

[15] Paaijmans K. Weather, water and malaria mosquito larvae, PhD Thesis, Wageningen University, The Netherlands. 2008; Available from: http://edepot.wur.nl/4348

[16] Gunda R, Chimbari MJ, Shamu S, Sartorius B, Mukaratirwa S (2017). Malaria incidence trends and their association with climatic variables in rural Gwanda, Zimbabwe, 2005–2015. Malar J.

[17] Ijumba J, Mosha F, Lindsay S (2002). Malaria transmission risk variations derived from different agricultural practices in an irrigated area of northern Tanzania. Med Vet Entomol.

[18] Amaechi EC, Ukpai OM, Ohaeri CC, Ejike UB, Irole-eze OP, Egwu O (2018). Distribution and seasonal abundance of Anopheline mosquitoes and their association with rainfall around irrigation and non-irrigation areas in Nigeria. UNED Res J.

[19] Ijumba J, Lindsay S (2001). Impact of irrigation on malaria in Africa : paddies paradox. Med Vet Entomol.

[20] Bhatt S, Weiss DJ, Cameron E, Bisanzio D, Mappin B, Dalrymple U (2016). The effect of malaria control on *Plasmodium falciparum* in Africa between 2000 and 2015. Nature.

[21] Tizifa TA, Kabaghe AN, Mccann RS, Van Den BH, Van VM, Phiri KS (2018). Prevention efforts for malaria. Curr Trop Med Reports.

[22] Nkumama IN, O’Meara WP, Osier FHA (2017). Changes in malaria epidemiology in Africa and new challenges for elimination. Trends Parasitol.

[23] Bødker R, Akida J, Shayo D, Kisinza W, Msangeni HA, Pedersen EM (2003). Relationship between altitude and intensity of malaria transmission in the Usambara Mountains. Tanzania J Med Entomol.

[24] Chen H, Githeko AK, Zhou G, Githure JI, Yan G (2006). New records of *Anopheles arabiensis* breeding on the Mount Kenya highlands indicate indigenous malaria transmission. Malar J.

[25] Alonso D, Bouma MJ, Pascual M (2011). Epidemic malaria and warmer temperatures in recent decades in an East African highland. Proc Soc Biol.

[26] Shekalaghe SA, Teun Bousema J, Kunei KK, Lushino P, Masokoto A, Wolters LR (2007). Submicroscopic *Plasmodium falciparum* gametocyte carriage is common in an area of low and seasonal transmission in Tanzania. Trop Med Int Health.

[27] Matowo J, Kitau J, Kabula B, Kavishe R, Oxborough R, Kaaya R (2014). Dynamics of insecticide resistance and the frequency of kdr mutation in the primary malaria vector *Anopheles arabiensis* in rural villages of Lower Moshi, North Eastern Tanzania. J Parasitol Vector Biol.

[28] Mahande A, Dusfour, IsabelleMatias, Jonathan R, Kweka E. Knockdown resistance, Rdl alleles, and the annual entomological inoculation rate of wild mosquito populations from Lower Moshi, Northern Tanzania. J Glob Infect Dis. 2012;4:114. 10.4103/0974-777X.96776 PMC3385201 22754247

[29] Essendi WM, Zalik AMV, Lo E, Machani MG, Zhou G, Githeko AK (2019). Epidemiological risk factors for clinical malaria infection in the highlands of Western Kenya. Malar J.

[30] Berzosa P, De LA, Barja MR, Herrador Z, González V, García L (2018). Comparison of three diagnostic methods (microscopy, RDT, and PCR ) for the detection of malaria parasites in representative samples from Equatorial Guinea. Malar J.

[31] Lee J, Jang W, Cho H, Kim Y, Han T, Yun G (2014). False-positive results for rapid diagnostic tests for malaria in patients with rheumatoid factor. J Clin Microbiol.

[32] Boyce RM, Muiru A, Reyes R, Ntaro M, Mulogo E, Matte M (2015). Impact of rapid diagnostic tests for the diagnosis and treatment of malaria at a peripheral health facility in Western Uganda : an interrupted time series analysis. Malar J.

[33] Lechthaler F, Id BM, Lechthaler-felber G, Likwela L, Mavoko HM, Rika JM (2019). Trends in reported malaria cases and the effects of malaria control in the Democratic Republic of the Congo. PLoS ONE.

[34] Malaria Operational Plan FY 2019. President’s Malaria Initiative, Tanzania. 2019; Available from: http://ihi.eprints.org/3314/1/Malaria_Strategic_Plan_Full_Version_02_27_14.pdf

[35] Ishengoma DS, Mmbando BP, Mandara CI, Chiduo MG, Francis F, Timiza W (2018). Trends of *Plasmodium falciparum* prevalence in two communities of Muheza district North-eastern Tanzania: correlation between parasite prevalence, malaria interventions and rainfall in the context of re-emergence of malaria after two decades of progressively declining transmission. Malar J.

[36] Newby H, Ivanovich E, Lynch M, Carvajal-Velez L, Bhattarai A, Cibulskis RE (2017). Framework for evaluating the health impact of the scale-up of malaria control interventions on all-cause child mortality in sub-Saharan Africa. Am J Trop Med Hyg.

[37] Musiime AK, Smith DL, Kilama M, Rek J, Arinaitwe E, Nankabirwa JI (2019). Impact of vector control interventions on malaria transmission intensity, outdoor vector biting rates and *Anopheles* mosquito species composition in Tororo. Uganda Malar J.

[38] Githinji S, Kistemann T. Insecticide treated nets; use, misuse or disuse. 13^th^ International Congress on Infectious Diseases Abstracts. Institute for Hygiene and Public Health University of Bonn, Bonn, Germany. Available at https://www.ijidonline.com/article/S1201-9712(08)00623-1/abstract.

[39] Berthe S, Harvey SA, Lynch M, Koenker H, Jumbe V, Khangamwa BK (2019). Poverty and food security: drivers of insecticide-treated mosquito net misuse in Malawi. Malar J.

[40] Khagayi S, Desai M, Amek N, Were V, Onyango ED, Odero C (2019). Modelling the relationship between malaria prevalence as a measure of transmission and mortality across age groups. Malar J.

[41] Mawili Mboumba DP, Bouyou-Akotet MK, Kendjo E, Nzamba J, Medang MO, Mbina JR (2013). Increase in malaria prevalence and age of at risk population in different areas of Gabon. Malar J.

[42] Desai M, ter Kuile FO, Nosten F, McGready R, Asamoa K, Brabin B (2007). Epidemiology and burden of malaria in pregnancy. Lancet Infect Dis.

[43] Mazigo HD, Rumisha SF, Chiduo MG, Bwana VM, Mboera LEG (2017). Malaria among rice farming communities in Kilangali village, Kilosa district, Central Tanzania: prevalence, intensity and associated factors. Infect Dis Poverty.

[44] Rolfes MA, McCarra M, Magak NG, Ernst KC, Dent AE, Lindblade KA (2012). Development of clinical immunity to malaria in highland areas of low and unstable transmission. Am J Trop Med Hyg.

[45] Garley A, Patton E, Eckert E, Negroustoueva S (2013). Gender differences in insecticide treated nets (ITN) use after a universal free distribution campaign in Kano State, Nigeria: post-campaign survey results. Malar J.

[46] Beck-Johnson LM, Nelson WA, Paaijmans KP, Read AF, Thomas MB, Bjørnstad ON (2013). The effect of temperature on *Anopheles* mosquito population dynamics and the potential for malaria transmission. PLoS ONE.

[47] Okorie N, Popoola K, Awobifa O, Ibrahim K, Ademowo GO (2014). Species composition and temporal distribution of mosquito populations in Ibadan. Southwest Nigeria J Entomol Zool Stud.

[48] Farajzadeh M, Halimi M, Ghavidel Y, Delavari M (2015). Spatiotemporal *Anopheles* population dynamics, response to climatic conditions: The case of Chabahar, South Baluchistan. Iran Ann Glob Health.

[49] Mabaso MLH, Craig M, Ross A, Smith T (2007). Environmental predictors of the seasonality of malaria transmission in Africa:the challenge. Am J Trop Med Hyg.

